# Dr. M'Whister, on Cancer

**Published:** 1802-01

**Authors:** Thomas M'Whister

**Affiliations:** Et Societ. Med. Phys. Edin. Soc. Hon. Newcastle-upon-Tyne


### *To the Editors of the Medical and Phyſical Journal.*

Gentlemen,

The caſe of Mr. Knight, of Friendſbury, communicated to you by Mr. Atkinſon, and inſerted in your laſt Journal, has rouſed my endeavours to recollect that of Mrs. Rowe. — Her diſeaſe, whether cancerous or not, having been deemed incurable, though it ultimately terminated favourably, is a circumſtance which may, perhaps, afford ſome comfort to your correſpondent with regard to his friend. In addition to the remedies mentioned before, I may inſert powdered chalk, which, according to the experience of ſeveral reſpectable practitioners here, has proved a very effectual application to ulcers both of the common and the cancerous kind. I regret that I have not been able to recollect ſeveral important circumſtances relative to Mrs. Rowe's caſe; but if, conſidering it as an outline, rather than a perfect repreſentation, you will favour it with a place in your valuable publication, I ſhall conſider it as honoured beyond its deſerts.

I am, &c.

THOMAS M'WHISTER, M. D.

Et Societ. Med. Phyſ. Edin. Soc. Hon.

*Newcaſtle-upon-Tyne, Dec.* 7, 1801.

---

Mrs. Rowe, an object equally of poverty and diſeaſe, conſulted me in ſpring laſt, on account of complaints apparently cancerous in their nature. She was a middle-aged woman of a very firm mind; and, previous to her illneſs, had been of an excellent conſtitution. Poverty compelled her to ſuckle her infant, then ſeveral months old, which did not ſeem conſtitutionally diſeaſed, although occaſionally ſickiſh, owing, perhaps, to a vitiated ſtate of its mother's milk. Both the breaſts of this poor woman were morbidly affected. The left one had an induration at its bafe, about the fize of an egg, and fuffered thofe frequent lancinating pains characteriftic of incipient cancer; while the right was hard, livid, immoveable, and raggedly fiftulous in feveral places, from which iffued fœtid, bloody, and corrofive matter. The former fupplied the child with milk, but the latter had loft the papilla, and no longer fecreted that fluid. According to the emphatic defcription of my patient, the pain of this breaft refembled "the frequent darting of fire through her flefh." The pains of the right breaft fometimes fhot into its fellow, and often towards the axillary glands of the fame fide, which were more difeafed than thofe of the other. But befides, pains analogous to the above affailed her uterus, accompanied by a difcharge of fœtid fanies from her pudenda, which excoriated the neighbouring parts; and hence it feemed probable that a relation fubfifted between thefe and her other complaints.

Under a combination of fuch calamities, with extreme poverty, her bodily vigour had confiderably declined, though it ftill enabled her to walk a few miles from the country, to vifit me once in the week. Her pulfe was generally quicker than natural, but particularly in the evening, when fhe occafionally became feverifh. Her appetite was much impaired, and fhe refted ill at night. After fevere fits of her pains fhe fometimes experienced faintnefs, fucceeded by naufea, fhiverings, and cold fweats. Such was the fituation of my patient at her firft vifit to me. How long fhe had been fo held, previoufly, I cannot precifely fay; but I am led to think fhe told me her complaints had then continued for feveral months, and had commenced fome time prior to the birth of her child. She had, however, confulted feveral practitioners in Maidftone, before my arrival there; they had humanely affifted her; but confidered her diforder to be a cancer, too deeply rooted to admit of eradication, even by the fcalpel.

It feemed poffible, notwithftanding, that we might all have miftaken the real nature of this cafe; and the encouragement of fuch a hope was, at any rate, calculated to increafe the perfeverance both of the patient and the phyfician.

With a view to promote a more healthy action in her breafts, ʒj. ungt. hyd. fort. was rubbed into them daily; and poultices compofed of bread and milk, or of the recent leaves of cicuta, were applied to that which was ulcerated. Injections of alum, diffolved in decoction of oak-bark, and occafional opiates, were alfo employed. Having followed this plan for about three weeks, her mouth became flightly affected by the mercury, but with little amendment of her frame. About this period I put her into the way of procuring a diet of rice and milk, inftead of of bread and tea, which had before been her only nourishment.

Continuing the moderate effects of her former medicines she now took two grains of pulv. fol. cicutæ, and three or four of opium in the day: these were formed into pills, one of which she used every two or three hours, or according to the urgency of her symptoms.

The quantity of the cicuta was gradually increased to six or eight grains in the day, but without manifesting any distinct operation; and that of the other remedies was regulated by their effects upon her system. The mercury never produced salivation, nor the opium and cicuta, aught but the alleviation of pain. From the time she began to take the pills till she laid them aside, a regular amendment was evident. Towards the close of the second month of her attendance upon me she began to disuse all her medicines by degrees; and very shortly afterwards took her final leave of me, seemingly without any ailment.

But, although I confess I am satisfied that a considerable portion of healing power was produced by the remedies exhibited to my patient, yet I think it my duty to own, that I am convinced she was greatly indebted also for her recovery to the attentions of some families in Maidstone, and particularly to the sisters of a neighbouring nobleman, noted alike on account of the extensive comfort they diffuse around them, and their judicious mode of administering it to the poor and to the distressed.

